# Targeting Dendritic Cells with Antigen-Delivering Antibodies for Amelioration of Autoimmunity in Animal Models of Multiple Sclerosis and Other Autoimmune Diseases

**DOI:** 10.3390/antib9020023

**Published:** 2020-06-15

**Authors:** Courtney A. Iberg, Daniel Hawiger

**Affiliations:** Department of Molecular Microbiology and Immunology, Saint Louis University School of Medicine, Doisy Research Center, 1205 Carr Lane, St. Louis, MO 63104, USA; courtney.iberg@slu.edu

**Keywords:** dendritic cells, tolerance, antigen targeting, chimeric antibodies, autoimmunity, multiple sclerosis, diabetes

## Abstract

The specific targeting of dendritic cells (DCs) using antigen-delivering antibodies has been established to be a highly efficient protocol for the induction of tolerance and protection from autoimmune processes in experimental autoimmune encephalomyelitis (EAE), a model of multiple sclerosis (MS), as well as in some other animal disease models. As the specific mechanisms of such induced tolerance are being investigated, the newly gained insights may also possibly help to design effective treatments for patients. Here we review approaches applied for the amelioration of autoimmunity in animal models based on antibody-mediated targeting of self-antigens to DCs. Further, we discuss relevant mechanisms of immunological tolerance that underlie such approaches, and we also offer some future perspectives for the application of similar methods in certain related disease settings such as transplantation.

## 1. Introduction

Over one hundred years ago, Paul Ehrlich coined the term “horror autotoxicus” to define an immune attack against an organism’s healthy tissues [1]. Since then, our knowledge of the complex mechanisms of the immune system as well as our understanding of the pathogenesis of specific autoimmune diseases have grown tremendously. However, despite this progress, autoimmunity continues to frustrate therapeutic efforts [2,3]. The mechanisms underlying the pathogenesis of the various autoimmune processes are complex, and they may result in a plethora of different morbidities, often displaying severe, debilitating symptoms. Such autoimmune diseases (including multiple sclerosis (MS) and type 1 diabetes) have a severe impact on the well-being of the afflicted individuals and also inevitably lead to broad socioeconomic costs [4,5]. The available treatments against autoimmune diseases fall into two main categories: restoration of functions that were lost as a result of specific disease processes (such as after a loss of nervous tissues or islet cells in MS or autoimmune diabetes, respectively) and prevention of further tissue damage by blocking the underlying aberrant functions of the immune system. Since the long-term success of any specific therapeutic approaches ultimately depends on limiting the underlying autoimmune process, immunotherapies are at the forefront of necessary treatments. However, currently available immunotherapy protocols are only partially effective. Further, by lacking targeting specificity, these approaches are often burdened by side effects. This necessitates the development of new approaches for immunomodulation that specifically aim to alter the functions of offending lymphocytes [6,7]. By developing new approaches based on the targeting of specific antigens for presentation by dendritic cells (DCs) that are able to elicit effective mechanisms of immunoregulation, one might envision treatments that selectively block the autoimmune process without affecting beneficial immune responses.

## 2. Utilizing Induction of Tolerance Through Antigen Targeting to DCs for Protection Against Autoimmunity

The common origins of most autoimmune responses depend on the aberrant activation of self-reactive T cells remaining in the mature repertoire of peripheral lymphocytes [8]. Such presence of self-reactive T cells is not necessarily a result of specific defects during the process of thymic selection. In fact, almost all T cell receptors (TCRs) are cross-reactive to some degree and can recognize multiple, sometimes even unrelated, peptides (known as “molecular mimics”) presented by major histocompatibility complex (MHC) molecules, therefore expanding the useful T cell repertoire against external and internal threats. However, these processes also inevitably increase the risk of some peripheral T cells being reactive against self-antigens [9,10,11,12,13,14]. This self-reactivity is partially attributed to the insufficient thymic deletion of T cells that are specific for tissue restricted antigens (TRAs). Such TRAs can trigger autoimmune responses when they are presented by specialized antigen presenting cells (APCs) in the peripheral immune system more efficiently than they are during their initial presentation to developing thymocytes by medullary thymic epithelial cells (mTECs) [15].

The autoimmune activation of self-reactive T cells is usually avoided by complex mechanisms of peripheral tolerance, including the crucial roles of thymically produced regulatory T cells (tTreg cells) [16]. However, even in the presence of tTreg cells, specific self-reactive peripheral T cells can still be primed by low-affinity self-antigens [13,14,15,17]. For example, in various relevant disease models, the autoimmune process can be initiated through the priming of pre-existing self-reactive T cells after the immunization of healthy animals with specific self-antigens in the context of an introduced infectious agent or in the presence of adjuvants [14,18]. These results suggest that a specific pro-inflammatory autoimmune activation can overwhelm the functions of tTreg cells. In some individuals, genetic dispositions also further contribute to an autoimmune process by compromising the specific development and functions of tTreg cells [13,14,15,19].

However, additional, extrathymically-induced mechanisms of antigen-specific tolerance help to control the activation and functions of autoreactive T cells and to maintain immune homeostasis. These mechanisms include crucial functions of peripherally induced Treg cells (pTreg cells) that may be induced by specialized DCs, which function as APCs critical for the initiation and regulation of T cell responses to foreign and self-antigens [20,21,22]. Some literature refers to such peripherally induced Treg cells as induced Treg cells (iTreg cells), as these cells are induced to increase their expression of the transcription factor forkhead box P3 (Foxp3). Currently, the term “iTreg cell” is usually used to refer to Treg cells that are induced from naïve CD4^+^ T cells In Vitro and that may also be subsequently transferred In Vivo to elicit desired responses [18,23,24,25]. In contrast, antigen-specific pTreg cells are induced In Vivo, and the availability of peripheral antigens is the sine qua non to a generation of such corresponding pTreg cells by DCs [18]. Generally, DCs efficiently collect and present to T cells diverse antigens including those from apoptotic materials derived from normal tissues [26]. However, antigens originating from organs that are insulated from the immune system under steady-state conditions, defined by the absence of specific pro-inflammatory signals, are also less readily available to commence the initial pTreg cell conversion, resulting in an overall weaker prevention of the subsequent specific autoimmune responses [18]. Therefore, novel methods that were devised to increase the availability of such specific self-antigens to pTreg cell-inducing DCs via their targeted delivery help to enhance the relevant mechanisms of peripheral tolerance.

In the absence of specific acute pro-inflammatory stimuli (“steady state”), DCs generally promote T cell tolerance that crucially relies on the induction of pTreg cells [18,27,28]. However, not all DCs are equally capable of inducing pTreg cells, and some DCs may inevitably exacerbate the disease state by priming autoimmune T cells [29]. The outcomes of antigen-specific interactions between DCs and T cells are governed by multiple factors including immunomodulatory molecules expressed by each cell type [22,30]. Conventional DCs (cDCs) and plasmacytoid DCs (pDCs) are the two main populations of DCs found in both humans and mice. cDCs and pDCs, which develop from bone marrow progenitors, differentiate into various subsets in multiple tissues, and the total cDC population may be further divided into the cDC1 and cDC2 subsets, which are characterized by some degree of specialization [22,29,31,32]. The transcription factors interferon regulatory factor 8 (Irf8), inhibitor of DNA-binding 2 (Id2), and basic leucine zipper transcription factor AFT-like 3 (Batf3) govern the development of cDC1s, and DCs of this subset can be distinguished by the expression of the X-C motif chemokine receptor 1 (XCR1). Importantly, some cDC1s also express B and T lymphocyte associated (BTLA). In contrast, the transcription factor interferon regulatory factor 4 (Irf4) governs the development of cDC2s; DCs of this subset are distinguished by the expression of CD172a (signal regulatory protein alpha (SIRPα)) [22,29,32,33].

Although the developmental designation of DC subsets does not strictly overlap with distinct immune functions, the specific subsets may be further characterized by a degree of functional specialization. Whereas cDC2s can preferentially promote Th2, Th17, and follicular helper T cell differentiation, cDC1s have crucial roles in the cross-priming of CD8^+^ T cells, the priming of Th1 cells, and, also, in the induction of CD4^+^CD25^+^Foxp3^+^ pTreg cells [21,29,30,34]. Therefore, a specific delivery of antigens intended for stimulating pTreg cell conversion needs to be crucially targeted towards DCs with the strongest capabilities to induce pTreg cells or to DCs that can expand other Treg cell populations. Due to their inherent tolerogenic properties based on a constitutive expression of key immunomodulatory molecules that govern tolerance (including BTLA, programmed death ligand-1 (PD-L1), the T cell costimulatory ligand B7h, and CD80/CD86, as well as cytokines such as transforming growth factor beta (TGF-β) and interleukin-10 (IL-10)), cDC1s are particularly good inducers of pTreg cells [18,21,22,35]. Consequently, such BTLA^hi^XCR1^+^CD172a^-^ cDC1s with specific tolerogenic functions have also been referred to as natural tolerogenic DCs (ntDCs), and, in addition to their high expression of BTLA, they also express DEC-205 [22].

Recent studies elucidated the specific mechanisms mediated by BTLA and its receptor, herpesvirus entry mediator (HVEM), expressed in naïve T cells, that activate the ETS1 transcription factor, leading to the increased expression of *Cd5* in such T cells [33,35]. The roles of CD5 in governing tolerance were initially considered in relation to its functions in the negative regulation of TCR signaling [36,37,38,39]. More recent studies, however, established that an increased expression of CD5 in CD4^+^ T cells specifically facilitates pTreg cell conversion by modulating a resistance to effector-differentiating cytokines [40]. Therefore, the specific upregulation of the expression and functions of CD5 in T cells by BTLA^hi^ ntDCs represents a key immunomodulatory mechanism operating complementarily to other pathways dependent on PD-L1/programmed cell death protein 1 (PD-1), CD80/CD86/cytotoxic T lymphocyte antigen 4 (CTLA-4), and B7h/inducible T cell costimulator (ICOS), which directly induce *Foxp3* expression in developing pTreg cells [18,30,35].

**Figure 1:**
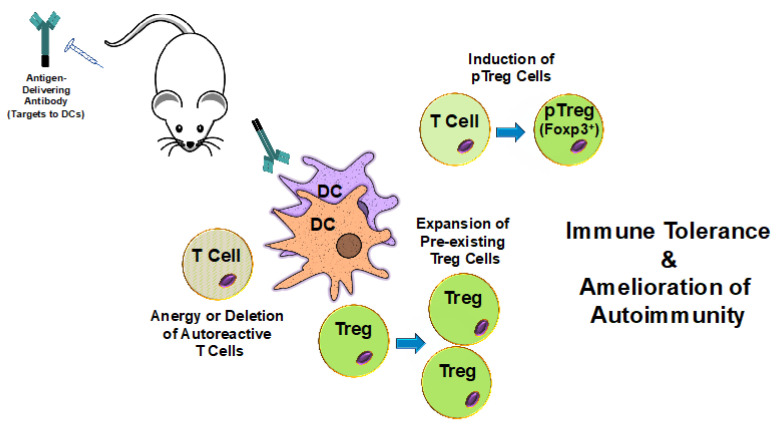
The delivery of self-antigens to dendritic cells induces tolerance and ameliorates autoimmunity. Antibodies specific for cell surface molecules expressed by dendritic cells (DCs) are fused with or conjugated to self-antigens. Upon In Vivo administration, these antibodies target the antigens to DCs. DCs then internalize, process, and present the delivered antigens to T cells. Natural tolerogenic DCs (ntDCs) are good inducers of peripheral regulatory T cells (pTreg cells) and are often selected for antigen targeting purposes. This results in the induction of pTreg cells and, ultimately, in immune tolerance to the specific self-antigens and amelioration of autoimmune disease symptom severity. Additionally, antigens presented by some tolerance-inducing DCs may also promote the expansion of pre-existing regulatory T cells (Treg cells) as well as the anergy or deletion of autoreactive T cells.

**Figure 2:**
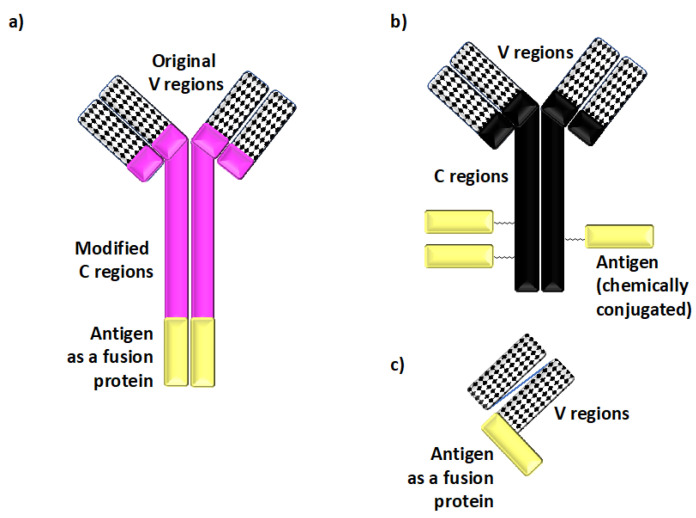
Defined antigens are delivered to dendritic cells In Vivo using recombinant chimeric and other types of antibodies. (**a**) Recombinant chimeric antibodies, which deliver defined peptide or protein antigens (shown in yellow in panels (a–c)) to specific dendritic cell (DC) cell surface molecules, are comprised of the variable (V) regions derived from monoclonal antibodies specific for cell surface molecules expressed on DCs and the species-specific heavy and light constant (C) regions derived from separate immunoglobulins. The peptide antigen of choice is genetically fused to the C regions. This recombinant chimeric antibody design enhances the targeting specificity In Vivo by minimizing non-specific binding to Fc receptors, and it also helps to avoid stoichiometric differences in the amounts of antigenic materials present in such reagents. (**b**) Antibody–antigen conjugates are comprised of antigenic proteins chemically conjugated to native antibodies specific for cell surface molecules expressed on DCs. Such conjugates have been successfully used to deliver defined antigens to DCs, although they may lack some of the targeting specificity-enhancing modifications found in recombinant chimeric antibody designs. (**c**) Single-chain fragment variable (scFv) constructs provide yet another means of delivering antigen In Vivo. scFv constructs are comprised of a linker joining the corresponding V regions genetically fused to the antigen for targeting.

Given the preponderance of specific molecules present on DCs with tolerogenic functions, the use of monoclonal antibodies has proven particularly successful among different methods of antigen delivery to direct antigens to ntDCs with defined tolerogenic properties [7,21,41] (Figure 1). Two major types of antigen-delivering antibodies have emerged: chimeric antibodies containing antigenic polypeptides as fusion proteins within the constant regions of recombinantly-modified immunoglobulins; and chemical conjugates between native antibodies and antigenic proteins [7] (Figure 2).

The recombinant chimeric antibodies applied the general design originally developed for the anti-DEC-205 chimeric antibody [42]. Most importantly, the original constant regions are replaced with engineered species-specific constant regions, which may include additional mutations introduced to minimize their non-specific binding to Fc receptors. Overall, in addition to allowing for a better specificity of targeting In Vivo, the use of such chimeric immunoglobulin fusion proteins also helps to avoid unintentional stoichiometric differences in the amounts of antigenic molecules present in these DC-targeting reagents [7,42].

Because of the strong pro-tolerogenic properties of DEC-205^+^BTLA^hi^ ntDCs, it is not surprising that antigen delivery methods based on targeting through DEC-205 have been successfully utilized for the induction of tolerance [7,21,22]. The originally developed approach based on an antigenic delivery through DEC-205 was subsequently extended to target other molecules expressed on DCs. Particularly, in addition to DEC-205, Langerin (CD207), Trem-like 4 (Treml4), and DC NK lectin group receptor-1/C-type lectin domain family 9A (DNGR-1/CLEC9A) have also been utilized for targeting antigens to some cDC1s, whereas dendritic cell inhibitory receptor 2 (DCIR2) has been used to target cDC2s [43,44,45,46,47,48,49,50,51,52]. As reviewed in [7], targeting antigens to the transmembrane protein Langerin, the cell surface receptor Treml4, or the C-type lectin domain family member CLEC9A has been shown to elicit antigen presentation to both CD4^+^ and CD8^+^ T cells, helping to alter disease severity in some models of autoimmune disease. As mentioned below, although cDC2s are less potent inducers of pTreg cells and tolerance, the targeting of antigens through DCIR2 present on these DCs has still been utilized for various types of immunomodulation and immunotherapies, and such targeting to DCIR2 has been shown to further increase the therapeutic potential of antigen targeting to DCs in a wide range of immune-mediated diseases and conditions [7,49,53]. Further, delivering antigens through Siglec-H and bone marrow stromal cell antigen 2 (BST2), both present on pDCs, further helped to extend the range of therapeutically relevant applications [47,48].

In addition to proteins that are expressed on specific DC subsets, the CD11c integrin, which is expressed by all murine cDCs, has also been targeted with antigens by various strategies In Vivo [54,55,56]. More recently, the recombinant chimeric anti-CD11c antibody was produced based on the design containing murine immunoglobulin IgG1 constant regions, as in the case of the original chimeric anti-DEC-205 antibody [33]. Since anti-CD11c delivers antigens to all DCs, irrespectively of subsets, the In Vivo administration of this chimeric antibody has been applied in genetically modified mice that lack specific subsets of DCs to further advance the understanding of the functions of individual cDC1 and cDC2 subsets in the immunoregulation of autoimmune responses [21,33].

The specific functions of DCs also depend on the localization of such DCs within the organism as well as on the overall immunological context of DC functions. Consistent with the emerging concept of “homeostatic maturation” [57,58,59,60], many DCs present in the peripheral lymphoid organs do not necessarily remain as “immature” immunological bystanders even in the steady state. Instead, some DCs can still activate T cells, but this outcome of T cell activation by ntDCs can result in the induction of pTreg cells and tolerance (as discussed above) [21,22,27]. Hence, these inherent tolerogenic functions provide a very strong rationale for the development of approaches seeking to enhance the antigen-specific immunoregulatory mechanisms mediated by ntDCs that reside in the peripheral immune system and that may remain unaffected by the developing autoimmune process that is limited to a specific site or organ. The induced pTreg cells or expanded Treg cells can then not only block the priming of effector T cells in the periphery but can also disseminate throughout multiple specific anatomical sites, helping to ameliorate the localized autoimmune responses mediated by antigen-specific effectors. In addition to relying on the inherent tolerogenic functions of ntDCs, it may also be possible to modify the functions of DCs present in localized pro-inflammatory settings. Multiple pathways that can potentially induce tolerogenic functions in DCs under pro-inflammatory conditions have been described, as we recently reviewed [22]. However, at present, it remains challenging to deliver antigens specifically to DCs residing in defined anatomical locations and with induced tolerogenic functions under generally pro-inflammatory conditions, and further research is necessary to achieve this goal [7]. Moreover, as reviewed in [61], the administration of DC vaccine formulations employing carriers with specific physical properties (including molecular size, pH, charge, or the inclusion of particular chemokines, cytokines, or adjuvants) can elicit desired tolerogenic or pro-immunogenic responses of DCs in peripheral lymphoid organs (including draining lymph nodes), although further work is necessitated to determine which specific carrier properties work best with specific delivery methods and are most appropriate for specific diseases and conditions.

## 3. Antigen Targeting to DCs in Models of MS

The initial studies that paved the way for using antigen-delivering chimeric antibodies to ameliorate autoimmunity were performed in an animal model of MS [41]. The mechanisms underlying the autoimmune process in MS crucially involve activation of autoreactive encephalitogenic T cells that attack components of the myelin sheath that surround the neuronal axons of the nerves of the central nervous system (CNS), leading to severe neurological symptoms [62,63]. Therefore, therapeutic approaches focused on limiting the activation of such self-reactive T cells are expected to play an important role in mitigating the specific autoimmune process in MS [18,62,63]. While the specific pathways leading to the activation of encephalitogenic T cells remain unclear, it is expected that neuronal antigens present in the periphery may facilitate the initial priming of T cells that subsequently migrate into the CNS and cause damage upon their reactivation [62,63]. Many crucial studies investigating possible new treatments of MS have been carried out by inducing an activation of such self-reactive T cells in an animal model of autoimmune CNS disease, experimental autoimmune encephalomyelitis (EAE), which, in many ways, mimics progressive and relapsing–remitting forms of MS. Acutely progressing EAE is induced by immunization with neuronal antigens such as myelin oligodendrocyte glycoprotein (MOG), whereas immunization with proteolipid protein (PLP) leads to the initiation of relapsing–remitting EAE [18,64,65,66,67,68]. Overall, the characteristic inflammation seen in EAE (including perivascular CD4^+^ T cell and mononuclear cell infiltrations and clinical presentation with ascending paralysis) makes EAE a relevant model for MS and a powerful model with which to study T cell-dependent autoimmunity [69,70]. Particularly, these experimental models offer an opportunity to study the impact of specific immune regulation on various mechanisms that cumulatively contribute to a neuroimmune disease process [18].

Very early experiments showed that EAE can be effectively prevented by the pre-administration of neuronal antigens in the non-inflammatory context [71]. Other early work further established that lymph node cells transferred from rats that were treated with myelin basic protein (MBP) administered in the absence of pro-inflammatory adjuvants protected the recipient rats from subsequently induced EAE, indicating an induction of a dominant tolerance presently attributed to the functions of Treg cells [18,72]. Subsequently, additional lines of investigation established that various forms of antigens derived from mouse spinal cord homogenates for their subsequent presentation In Vivo in a non-inflammatory context, as well as various purified myelin-derived peptides, were able to confer immune tolerance that prevented subsequently induced EAE [73,74,75,76].

In contrast to such treatments that lacked specificity in terms of the types of APCs responsible for presentation of antigens to T cells, approaches based on the targeting of antigens using antibodies specific for defined types of DCs allowed for the presentation of antigens in a specific tolerogenic context. In the initial experiments, the treatment with an anti-DEC-205 chimeric antibody fused with the MOG_35–55_ peptide resulted in a tolerogenic activation of MOG-specific T cells and, ultimately, in tolerance that prevented both the symptoms of subsequently induced EAE and the accumulation of encephalitogenic T cells in the CNS [41]. The capacity for effective immunomodulation and amelioration of the symptoms of EAE via a targeted delivery of specific antigens to DCs was further extended in subsequent studies that included the delivery of MOG to pDCs; in such studies, MOG targeting prevented subsequently induced EAE, and an expansion of Treg cells was suggested as a possible mechanism [47]. Similarly, the delivery of MOG to Langerin^+^ migratory DCs proved efficacious in blocking specific autoimmune responses [44]. Single-chain fragment variable (scFv) constructs specific for DEC-205 and fused to MOG were also used to induce specific tolerance [26,77,78]. In addition to the immunomodulation achieved in acute MOG-mediated EAE, anti-DEC-205-mediated delivery of the specific PLP_139–151_ peptide also ameliorated clinical symptoms in the relapsing–remitting model of EAE [79]. Further, the use of anti-DCIR2 fusion proteins to target the PLP_139–151_ antigen to DCIR2^+^ cDC2s was efficacious in the amelioration of EAE symptom severity in the relapsing–remitting model through complex mechanisms, possibly including the expansion of pre-existing Treg cell populations [53].

Multiple previous studies established the crucial roles of Foxp3^+^ Treg cells in the protection and recovery from MS and EAE and showed that the absence of Treg cells or their abnormal functions exacerbated the severity of disease both in patients and in animal models of MS [80,81,82,83,84,85,86,87,88,89,90,91,92,93]. These results extended the rationale for Treg cell-mediated therapeutic approaches that was initially based on the early findings that the course of disease could be mitigated by Treg cells actively arising during the recovery stage of EAE [94,95,96,97]. However, in the early stages of MS, many patients have the same frequency of Treg cells in their peripheral blood [86,92]. Similarly, the normal presence of a Treg cell population predominantly consisting of tTreg cells in healthy animals cannot prevent the development of EAE upon specific immunization [18]. Therefore, an induction of specific pTreg cells is indispensable for the suppression of disease, and the de novo induction of pTreg cells was established as a fundamental mechanism of long-term tolerance and protection from EAE induced by antigens from nervous tissues that may be processed and presented to peripheral T cells by DCs [18,28]. However, in healthy organisms, such antigens originating from the CNS usually remain insulated from the immune system. Correspondingly, the numbers of neuronal antigen-specific pTreg cells are small, but such pTreg cells can be further induced by the increased presence of specific peripheral antigens, made available via antibody-mediated targeting In Vivo [18,28].

This DC-induced, pTreg cell-dependent tolerance is specifically abrogated in the absence of homeodomain only protein (Hopx), a transcription cofactor required for the normal survival of pTreg cells during an acute antigenic rechallenge under pro-inflammatory conditions [18,28,98]. Therefore, genetic deficiencies interfering with the normal conversion or functions of pTreg cells affect the course of the autoimmune process to the extent determined by the initial exposure to specific antigens promoting the formation of corresponding pTreg cells [18,28,99].

## 4. Antigen Targeting to DCs in Models of Other Autoimmune Diseases

The targeting of antigens to DCs has also been effective in inducing tolerance to prevent autoimmunity in models of other autoimmune diseases, such as type 1 diabetes. Type 1 diabetes, also referred to as “autoimmune diabetes”, is a chronic autoimmune disease characterized by spontaneous, aberrant T and B cell immune responses against pancreatic beta cells, whose physiological function is to secrete insulin to regulate glucose metabolism [100,101]. It has been suggested that, over time, such immune responses destroy the beta cells to the degree that they can no longer regenerate, thereby nearly eradicating homeostatic glucose metabolism. Type 1 diabetes remains a major threat to patients, as a specific cure is currently elusive and present treatment requires continuous glucose monitoring and strict insulin therapy [101].

Though the non-obese diabetic (NOD) mouse has been the primary animal model of spontaneous type 1 diabetes, important insights were also gained from studies using double transgenic mice, in which TCR transgenic CD4^+^ T cells are specific for hemagglutinin (HA), which is expressed as a neo-self antigen by pancreatic beta cells [102,103,104,105,106]. These studies employing the targeted delivery of HA chemically conjugated to an anti-DEC-205 antibody demonstrated that tolerance induced by such model islet-specific antigens could prevent disease development [105,107].

Correspondingly, other studies utilizing the highly relevant NOD model of spontaneous autoimmune diabetes demonstrated that the chimeric antibody-mediated delivery of beta cell or insulin antigens to various DC subsets resulted in the amelioration of ongoing autoimmune processes and disease severity through the deletion of antigen-specific CD4^+^ and CD8^+^ T cells, as well as through an additional conversion of some autoreactive T cells into Foxp3^+^ pTreg cells [108,109,110,111]. These studies also underscored that the chimeric antibody-mediated delivery of antigen was markedly more therapeutic than the administration of free synthetic peptide, which was actually found to accelerate disease progression [110]. Overall, similar to its efficacy in MS models, the targeting of antigens using antibodies is being considered as a possible, important therapeutic tool in the treatment of autoimmune diabetes [111].

Though MS and type 1 diabetes currently represent the autoimmune diseases that have primarily been implicated in studies involving antibody-mediated antigen targeting to tolerogenic DCs, this targeting method has also been applied to other diseases. For instance, the targeted delivery of antigens to DEC-205^+^ DCs has ameliorated disease severity in models of proteoglycan-induced arthritis, inflammatory bowel disease (IBD), and spontaneous experimental autoimmune uveoretinitis (EAU), a murine T cell-mediated model of ocular inflammation [112,113,114,115]. In such studies, decreased disease severity was also associated with localized increases in the numbers of tolerance-promoting CD4^+^CD25^+^Foxp3^+^ Treg cells. Interestingly, it was also found that, when antigen was targeted to DCIR2^+^ DCs, EAU development was augmented, characterized by localized reductions in Treg cells [115]. It is also interesting to speculate that the potential targeting of antigens to the small yet varied populations of DCs found in the retina (as reviewed in [116]) may one day provide treatments for some other ocular diseases sharing immune-related pathogenesis.

Although not a main focus of this review, inducing immune tolerance towards transplanted tissues remains a crucial scientific and therapeutic concern. In patients with end-stage organ failure, the transplantation of functional organs from living or deceased donors has been the treatment of choice for nearly seventy years; however, the ongoing threat of immune-mediated rejection of the grafted tissue remains dire, despite the lifelong administration of immunosuppressive drugs to tissue recipients [117]. Tolerance to the grafted tissue, often referred to as “transplantation tolerance”, occurs when a donor’s transplanted tissue is functioning properly in the recipient and the recipient’s fully functional immune system does not mount a pathogenic, destructive response to that tissue (particularly in the absence of immunosuppressive drugs) [118,119]. Various obstacles resisting broad-spectrum transplantation tolerance exist, including the lack of robust assays to measure tolerance, the translatability of findings from animal studies into treatments for human patients, and, arguably most importantly, the need to modulate the recipient’s T cell responses by amplifying tolerance to donor tissue alloantigens [118].

Notably, transplantation tolerance has been enhanced via the antibody-mediated delivery of antigens to DCs, as reviewed in [120,121]. For example, the administration of intact MHC molecules conjugated to anti-DCIR2 antibodies could promote the DC-mediated amelioration of alloresponses toward alloantigens [122,123]. Moreover, as the targeting of collagen antigen to DEC-205^+^ DCs has proven successful in the prevention of skin graft rejection, ongoing studies and clinical trials seek to understand the efficacy of tolerogenic DC therapy in kidney and liver transplant recipients by using various methods of DC modulation [124,125,126,127].

## 5. Conclusions

In conclusion, the induction and modulation of tolerance by targeting DCs with antigen-delivering antibodies have proven successful in the amelioration of disease processes in a range of animal models including MS and diabetes. It remains possible that the targeting of disease-specific antigens to DCs may also be used to alleviate symptom severity in other diseases and their models. Therefore, as research progresses, additional antigenic targets such as the ubiquitin–proteasome system and ubiquitin ligase proteins may be considered for use in treatments of specific autoimmune disease pathologies and in subsequent therapies involving antibody-mediated antigen targeting [128]. Most importantly, such therapies may eventually become available to patients afflicted with specific autoimmune diseases. As mentioned previously, many important similarities exist between human and mouse DCs, particularly with regards to functional specialization of DC subsets and cell surface molecule expression (including the expression of molecules that are commonly used for targeting). Therefore, such commonalities provide promise for future clinical translation and therapeutic application of the methods of antigen targeting to DCs, although additional basic and clinical research is necessary to clarify the specific functions of anti-disease tolerance promoted by such DCs [22,129,130].

## References

[1] Steinman R.M., Nussenzweig M.C. (2002). Avoiding horror autotoxicus: The importance of dendritic cells in peripheral T cell tolerance. Proc. Natl. Acad. Sci. USA.

[2] Horwitz D.A., Fahmy T.M., Piccirillo C.A., La Cava A. (2019). Rebalancing Immune Homeostasis to Treat Autoimmune Diseases. Trends Immunol..

[3] Mosanya C.H., Isaacs J.D. (2019). Tolerising cellular therapies: What is their promise for autoimmune disease?. Ann. Rheum. Dis..

[4] Calixto O.J., Anaya J.M. (2014). Socioeconomic status. The relationship with health and autoimmune diseases. Autoimmun. Rev..

[5] Roberts M.H., Erdei E. (2020). Comparative United States autoimmune disease rates for 2010–2016 by sex, geographic region, and race. Autoimmun. Rev..

[6] Liebman H.A. (2016). Immune modulation for autoimmune disorders: Evolution of therapeutics. Semin. Hematol..

[7] Iberg C.A., Hawiger D. (2019). Advancing immunomodulation by in vivo antigen delivery to DEC-205 and other cell surface molecules using recombinant chimeric antibodies. Int. Immunopharmacol..

[8] Richards D.M., Kyewski B., Feuerer M. (2016). Re-examining the Nature and Function of Self-Reactive T cells. Trends Immunol..

[9] Wucherpfennig K.W. (2004). T cell receptor crossreactivity as a general property of T cell recognition. Mol. Immunol..

[10] Nelson R.W., Beisang D., Tubo N.J., Dileepan T., Wiesner D.L., Nielsen K., Wuthrich M., Klein B.S., Kotov D.I., Spanier J.A. (2015). T cell receptor cross-reactivity between similar foreign and self peptides influences naive cell population size and autoimmunity. Immunity.

[11] Anderson A.C., Nicholson L.B., Legge K.L., Turchin V., Zaghouani H., Kuchroo V.K. (2000). High frequency of autoreactive myelin proteolipid protein-specific T cells in the periphery of naive mice: Mechanisms of selection of the self-reactive repertoire. J. Exp. Med..

[12] Bouneaud C., Kourilsky P., Bousso P. (2000). Impact of negative selection on the T cell repertoire reactive to a self-peptide: A large fraction of T cell clones escapes clonal deletion. Immunity.

[13] Koehli S., Naeher D., Galati-Fournier V., Zehn D., Palmer E. (2014). Optimal T-cell receptor affinity for inducing autoimmunity. Proc. Natl. Acad. Sci. USA.

[14] Zehn D., Bevan M.J. (2006). T cells with low avidity for a tissue-restricted antigen routinely evade central and peripheral tolerance and cause autoimmunity. Immunity.

[15] Enouz S., Carrie L., Merkler D., Bevan M.J., Zehn D. (2012). Autoreactive T cells bypass negative selection and respond to self-antigen stimulation during infection. J. Exp. Med..

[16] Josefowicz S.Z., Lu L.F., Rudensky A.Y. (2012). Regulatory T cells: Mechanisms of differentiation and function. Annu. Rev. Immunol..

[17] Richards D.M., Ruggiero E., Hofer A.C., Sefrin J.P., Schmidt M., von Kalle C., Feuerer M. (2015). The Contained Self-Reactive Peripheral T Cell Repertoire: Size, Diversity, and Cellular Composition. J. Immunol..

[18] Jones A., Hawiger D. (2017). Peripherally Induced Regulatory T Cells: Recruited Protectors of the Central Nervous System against Autoimmune Neuroinflammation. Front. Immunol..

[19] Ooi J.D., Petersen J., Tan Y.H., Huynh M., Willett Z.J., Ramarathinam S.H., Eggenhuizen P.J., Loh K.L., Watson K.A., Gan P.Y. (2017). Dominant protection from HLA-linked autoimmunity by antigen-specific regulatory T cells. Nature.

[20] Mellman I. (2013). Dendritic cells: Master regulators of the immune response. Cancer Immunol. Res..

[21] Iberg C.A., Jones A., Hawiger D. (2017). Dendritic Cells As Inducers of Peripheral Tolerance. Trends Immunol..

[22] Iberg C.A., Hawiger D. (2020). Natural and Induced Tolerogenic Dendritic Cells. J. Immunol..

[23] Josefowicz S.Z., Rudensky A. (2009). Control of regulatory T cell lineage commitment and maintenance. Immunity.

[24] Chen W., Jin W., Hardegen N., Lei K.J., Li L., Marinos N., McGrady G., Wahl S.M. (2003). Conversion of peripheral CD4^+^CD25- naive T cells to CD4^+^CD25^+^ regulatory T cells by TGF-beta induction of transcription factor Foxp3. J. Exp. Med..

[25] Abbas A.K., Benoist C., Bluestone J.A., Campbell D.J., Ghosh S., Hori S., Jiang S., Kuchroo V.K., Mathis D., Roncarolo M.G. (2013). Regulatory T cells: Recommendations to simplify the nomenclature. Nat. Immunol..

[26] Iyoda T., Shimoyama S., Liu K., Omatsu Y., Akiyama Y., Maeda Y., Takahara K., Steinman R.M., Inaba K. (2002). The CD8^+^ dendritic cell subset selectively endocytoses dying cells in culture and in vivo. J. Exp. Med..

[27] Kretschmer K., Apostolou I., Hawiger D., Khazaie K., Nussenzweig M.C., von Boehmer H. (2005). Inducing and expanding regulatory T cell populations by foreign antigen. Nat. Immunol..

[28] Jones A., Opejin A., Henderson J.G., Gross C., Jain R., Epstein J.A., Flavell R.A., Hawiger D. (2015). Peripherally Induced Tolerance Depends on Peripheral Regulatory T Cells That Require Hopx To Inhibit Intrinsic IL-2 Expression. J. Immunol..

[29] Durai V., Murphy K.M. (2016). Functions of Murine Dendritic Cells. Immunity.

[30] Bourque J., Hawiger D. (2018). Immunomodulatory Bonds of the Partnership between Dendritic Cells and T Cells. Crit. Rev. Immunol..

[31] Collin M., Bigley V. (2018). Human dendritic cell subsets: An update. Immunology.

[32] Guilliams M., Dutertre C.A., Scott C.L., McGovern N., Sichien D., Chakarov S., Van Gassen S., Chen J., Poidinger M., De Prijck S. (2016). Unsupervised High-Dimensional Analysis Aligns Dendritic Cells across Tissues and Species. Immunity.

[33] Jones A., Bourque J., Kuehm L., Opejin A., Teague R.M., Gross C., Hawiger D. (2016). Immunomodulatory Functions of BTLA and HVEM Govern Induction of Extrathymic Regulatory T Cells and Tolerance by Dendritic Cells. Immunity.

[34] Krishnaswamy J.K., Alsen S., Yrlid U., Eisenbarth S.C., Williams A. (2018). Determination of T Follicular Helper Cell Fate by Dendritic Cells. Front. Immunol..

[35] Bourque J., Hawiger D. (2019). The BTLA–HVEM–CD5 Immunoregulatory Axis—An Instructive Mechanism Governing pTreg Cell Differentiation. Front. Immunol..

[36] Tarakhovsky A., Kanner S.B., Hombach J., Ledbetter J.A., Muller W., Killeen N., Rajewsky K. (1995). A role for CD5 in TCR-mediated signal transduction and thymocyte selection. Science.

[37] Azzam H.S., Grinberg A., Lui K., Shen H., Shores E.W., Love P.E. (1998). CD5 expression is developmentally regulated by T cell receptor (TCR) signals and TCR avidity. J. Exp. Med..

[38] Perez-Villar J.J., Whitney G.S., Bowen M.A., Hewgill D.H., Aruffo A.A., Kanner S.B. (1999). CD5 negatively regulates the T-cell antigen receptor signal transduction pathway: Involvement of SH2-containing phosphotyrosine phosphatase SHP-1. Mol. Cell Biol..

[39] Azzam H.S., DeJarnette J.B., Huang K., Emmons R., Park C.S., Sommers C.L., El-Khoury D., Shores E.W., Love P.E. (2001). Fine tuning of TCR signaling by CD5. J. Immunol..

[40] Henderson J.G., Opejin A., Jones A., Gross C., Hawiger D. (2015). CD5 Instructs Extrathymic Regulatory T Cell Development in Response to Self and Tolerizing Antigens. Immunity.

[41] Hawiger D., Masilamani R.F., Bettelli E., Kuchroo V.K., Nussenzweig M.C. (2004). Immunological unresponsiveness characterized by increased expression of CD5 on peripheral T cells induced by dendritic cells in vivo. Immunity.

[42] Hawiger D., Inaba K., Dorsett Y., Guo M., Mahnke K., Rivera M., Ravetch J.V., Steinman R.M., Nussenzweig M.C. (2001). Dendritic cells induce peripheral T cell unresponsiveness under steady state conditions in vivo. J. Exp. Med..

[43] Idoyaga J., Cheong C., Suda K., Suda N., Kim J.Y., Lee H., Park C.G., Steinman R.M. (2008). Cutting edge: Langerin/CD207 receptor on dendritic cells mediates efficient antigen presentation on MHC I and II products in vivo. J. Immunol..

[44] Idoyaga J., Fiorese C., Zbytnuik L., Lubkin A., Miller J., Malissen B., Mucida D., Merad M., Steinman R.M. (2013). Specialized role of migratory dendritic cells in peripheral tolerance induction. J. Clin. Investig..

[45] Idoyaga J., Lubkin A., Fiorese C., Lahoud M.H., Caminschi I., Huang Y., Rodriguez A., Clausen B.E., Park C.G., Trumpfheller C. (2011). Comparable T helper 1 (Th1) and CD8 T-cell immunity by targeting HIV gag p24 to CD8 dendritic cells within antibodies to Langerin, DEC205, and Clec9A. Proc. Natl. Acad. Sci. USA.

[46] Hemmi H., Zaidi N., Wang B., Matos I., Fiorese C., Lubkin A., Zbytnuik L., Suda K., Zhang K., Noda M. (2012). Treml4, an Ig superfamily member, mediates presentation of several antigens to T cells in vivo, including protective immunity to HER2 protein. J. Immunol..

[47] Loschko J., Heink S., Hackl D., Dudziak D., Reindl W., Korn T., Krug A.B. (2011). Antigen targeting to plasmacytoid dendritic cells via Siglec-H inhibits Th cell-dependent autoimmunity. J. Immunol..

[48] Loschko J., Schlitzer A., Dudziak D., Drexler I., Sandholzer N., Bourquin C., Reindl W., Krug A.B. (2011). Antigen delivery to plasmacytoid dendritic cells via BST2 induces protective T cell-mediated immunity. J. Immunol..

[49] Dudziak D., Kamphorst A.O., Heidkamp G.F., Buchholz V.R., Trumpfheller C., Yamazaki S., Cheong C., Liu K., Lee H.W., Park C.G. (2007). Differential antigen processing by dendritic cell subsets in vivo. Science.

[50] Sancho D., Mourao-Sa D., Joffre O.P., Schulz O., Rogers N.C., Pennington D.J., Carlyle J.R., Reis e Sousa C. (2008). Tumor therapy in mice via antigen targeting to a novel, DC-restricted C-type lectin. J. Clin. Investig..

[51] Tacken P.J., de Vries I.J., Gijzen K., Joosten B., Wu D., Rother R.P., Faas S.J., Punt C.J., Torensma R., Adema G.J. (2005). Effective induction of naive and recall T-cell responses by targeting antigen to human dendritic cells via a humanized anti-DC-SIGN antibody. Blood.

[52] Joffre O.P., Sancho D., Zelenay S., Keller A.M., Reis e Sousa C. (2010). Efficient and versatile manipulation of the peripheral CD4^+^ T-cell compartment by antigen targeting to DNGR-1/CLEC9A. Eur. J. Immunol..

[53] Tabansky I., Keskin D.B., Watts D., Petzold C., Funaro M., Sands W., Wright P., Yunis E.J., Najjar S., Diamond B. (2018). Targeting DEC-205(-)DCIR2(+) dendritic cells promotes immunological tolerance in proteolipid protein-induced experimental autoimmune encephalomyelitis. Mol. Med..

[54] Castro F.V., Tutt A.L., White A.L., Teeling J.L., James S., French R.R., Glennie M.J. (2008). CD11c provides an effective immunotarget for the generation of both CD4 and CD8 T cell responses. Eur. J. Immunol..

[55] Chappell C.P., Giltiay N.V., Dresch C., Clark E.A. (2014). Controlling immune responses by targeting antigens to dendritic cell subsets and B cells. Int. Immunol..

[56] White A.L., Tutt A.L., James S., Wilkinson K.A., Castro F.V., Dixon S.V., Hitchcock J., Khan M., Al-Shamkhani A., Cunningham A.F. (2010). Ligation of CD11c during vaccination promotes germinal centre induction and robust humoral responses without adjuvant. Immunology.

[57] Vander Lugt B., Riddell J., Khan A.A., Hackney J.A., Lesch J., DeVoss J., Weirauch M.T., Singh H., Mellman I. (2017). Transcriptional determinants of tolerogenic and immunogenic states during dendritic cell maturation. J. Cell Biol..

[58] Ardouin L., Luche H., Chelbi R., Carpentier S., Shawket A., Montanana Sanchis F., Santa Maria C., Grenot P., Alexandre Y., Gregoire C. (2016). Broad and Largely Concordant Molecular Changes Characterize Tolerogenic and Immunogenic Dendritic Cell Maturation in Thymus and Periphery. Immunity.

[59] Jiang A., Bloom O., Ono S., Cui W., Unternaehrer J., Jiang S., Whitney J.A., Connolly J., Banchereau J., Mellman I. (2007). Disruption of E-cadherin-mediated adhesion induces a functionally distinct pathway of dendritic cell maturation. Immunity.

[60] Baratin M., Foray C., Demaria O., Habbeddine M., Pollet E., Maurizio J., Verthuy C., Davanture S., Azukizawa H., Flores-Langarica A. (2015). Homeostatic NF-kappaB Signaling in Steady-State Migratory Dendritic Cells Regulates Immune Homeostasis and Tolerance. Immunity.

[61] Leleux J., Atalis A., Roy K. (2015). Engineering immunity: Modulating dendritic cell subsets and lymph node response to direct immune-polarization and vaccine efficacy. J. Control. Release.

[62] Compston A., Coles A. (2008). Multiple sclerosis. Lancet.

[63] Dendrou C.A., Fugger L., Friese M.A. (2015). Immunopathology of multiple sclerosis. Nat. Rev. Immunol..

[64] McMahon E.J., Bailey S.L., Castenada C.V., Waldner H., Miller S.D. (2005). Epitope spreading initiates in the CNS in two mouse models of multiple sclerosis. Nat. Med..

[65] Smilek D.E., Gautam A.M., Pearson C., Steinman L., McDevitt H.O. (1992). EAE: A model for immune intervention with synthetic peptides. Int. Rev. Immunol..

[66] Mendel I., Kerlero de Rosbo N., Ben-Nun A. (1995). A myelin oligodendrocyte glycoprotein peptide induces typical chronic experimental autoimmune encephalomyelitis in H-2b mice: Fine specificity and T cell receptor V beta expression of encephalitogenic T cells. Eur. J. Immunol..

[67] Kuchroo V.K., Anderson A.C., Waldner H., Munder M., Bettelli E., Nicholson L.B. (2002). T cell response in experimental autoimmune encephalomyelitis (EAE): Role of self and cross-reactive antigens in shaping, tuning, and regulating the autopathogenic T cell repertoire. Annu. Rev. Immunol..

[68] Simmons S.B., Pierson E.R., Lee S.Y., Goverman J.M. (2013). Modeling the heterogeneity of multiple sclerosis in animals. Trends Immunol..

[69] Miller S.D., Karpus W.J. Experimental Autoimmune Encephalomyelitis in the Mouse. https://europepmc.org/article/pmc/pmc2915550.

[70] Miller S.D., Karpus W.J., Davidson T.S. Experimental Autoimmune Encephalomyelitis in the Mouse. https://pubmed.ncbi.nlm.nih.gov/20143314/.

[71] Svet-Moldavskaya I.A., Svetmoldavsky G.J. (1958). Acquired resistance to experimental allergic encephalomyelitis. Nature.

[72] Swierkosz J.E., Swanborg R.H. (1975). Suppressor cell control of unresponsiveness to experimental allergic encephalomyelitis. J. Immunol..

[73] Kennedy M.K., Tan L.J., Dal Canto M.C., Miller S.D. (1990). Regulation of the effector stages of experimental autoimmune encephalomyelitis via neuroantigen-specific tolerance induction. J. Immunol..

[74] Kennedy M.K., Tan L.J., Dal Canto M.C., Tuohy V.K., Lu Z.J., Trotter J.L., Miller S.D. (1990). Inhibition of murine relapsing experimental autoimmune encephalomyelitis by immune tolerance to proteolipid protein and its encephalitogenic peptides. J. Immunol..

[75] Vandenbark A.A., Celnik B., Vainiene M., Miller S.D., Offner H. (1995). Myelin antigen-coupled splenocytes suppress experimental autoimmune encephalomyelitis in Lewis rats through a partially reversible anergy mechanism. J. Immunol..

[76] Turley D.M., Miller S.D. (2007). Peripheral tolerance induction using ethylenecarbodiimide-fixed APCs uses both direct and indirect mechanisms of antigen presentation for prevention of experimental autoimmune encephalomyelitis. J. Immunol..

[77] Ahmad Z.A., Yeap S.K., Ali A.M., Ho W.Y., Alitheen N.B., Hamid M. (2012). scFv antibody: Principles and clinical application. Clin. Dev. Immunol..

[78] Ring S., Maas M., Nettelbeck D.M., Enk A.H., Mahnke K. (2013). Targeting of autoantigens to DEC205(+) dendritic cells in vivo suppresses experimental allergic encephalomyelitis in mice. J. Immunol..

[79] Stern J.N., Keskin D.B., Kato Z., Waldner H., Schallenberg S., Anderson A., von Boehmer H., Kretschmer K., Strominger J.L. (2010). Promoting tolerance to proteolipid protein-induced experimental autoimmune encephalomyelitis through targeting dendritic cells. Proc. Natl. Acad. Sci. USA.

[80] Kleinewietfeld M., Hafler D.A. (2014). Regulatory T cells in autoimmune neuroinflammation. Immunol. Rev..

[81] O’Connor R.A., Anderton S.M. (2008). Foxp3+ regulatory T cells in the control of experimental CNS autoimmune disease. J. Neuroimmunol..

[82] Lowther D.E., Hafler D.A. (2012). Regulatory T cells in the central nervous system. Immunol. Rev..

[83] Vahl J.C., Drees C., Heger K., Heink S., Fischer J.C., Nedjic J., Ohkura N., Morikawa H., Poeck H., Schallenberg S. (2014). Continuous T cell receptor signals maintain a functional regulatory T cell pool. Immunity.

[84] McHugh R.S., Shevach E.M. (2002). The role of suppressor T cells in regulation of immune responses. J. Allergy Clin. Immunol..

[85] Lafaille J.J., Nagashima K., Katsuki M., Tonegawa S. (1994). High incidence of spontaneous autoimmune encephalomyelitis in immunodeficient anti-myelin basic protein T cell receptor transgenic mice. Cell.

[86] Feger U., Luther C., Poeschel S., Melms A., Tolosa E., Wiendl H. (2007). Increased frequency of CD4^+^ CD25^+^ regulatory T cells in the cerebrospinal fluid but not in the blood of multiple sclerosis patients. Clin. Exp. Immunol..

[87] Haas J., Hug A., Viehover A., Fritzsching B., Falk C.S., Filser A., Vetter T., Milkova L., Korporal M., Fritz B. (2005). Reduced suppressive effect of CD4^+^CD25high regulatory T cells on the T cell immune response against myelin oligodendrocyte glycoprotein in patients with multiple sclerosis. Eur. J. Immunol..

[88] Kumar M., Putzki N., Limmroth V., Remus R., Lindemann M., Knop D., Mueller N., Hardt C., Kreuzfelder E., Grosse-Wilde H. (2006). CD4^+^CD25^+^FoxP3^+^ T lymphocytes fail to suppress myelin basic protein-induced proliferation in patients with multiple sclerosis. J. Neuroimmunol..

[89] Venken K., Hellings N., Broekmans T., Hensen K., Rummens J.L., Stinissen P. (2008). Natural naive CD4^+^CD25^+^CD127low regulatory T cell (Treg) development and function are disturbed in multiple sclerosis patients: Recovery of memory Treg homeostasis during disease progression. J. Immunol..

[90] Venken K., Hellings N., Hensen K., Rummens J.L., Medaer R., D’Hooghe M.B., Dubois B., Raus J., Stinissen P. (2006). Secondary progressive in contrast to relapsing-remitting multiple sclerosis patients show a normal CD4^+^CD25^+^ regulatory T-cell function and FOXP3 expression. J. Neurosci. Res..

[91] Frisullo G., Nociti V., Iorio R., Patanella A.K., Caggiula M., Marti A., Sancricca C., Angelucci F., Mirabella M., Tonali P.A. (2009). Regulatory T cells fail to suppress CD4T+-bet+ T cells in relapsing multiple sclerosis patients. Immunology.

[92] Venken K., Hellings N., Thewissen M., Somers V., Hensen K., Rummens J.L., Medaer R., Hupperts R., Stinissen P. (2008). Compromised CD4^+^ CD25(high) regulatory T-cell function in patients with relapsing-remitting multiple sclerosis is correlated with a reduced frequency of FOXP3-positive cells and reduced FOXP3 expression at the single-cell level. Immunology.

[93] Kohm A.P., McMahon J.S., Podojil J.R., Begolka W.S., DeGutes M., Kasprowicz D.J., Ziegler S.F., Miller S.D. (2006). Cutting Edge: Anti-CD25 monoclonal antibody injection results in the functional inactivation, not depletion, of CD4^+^CD25^+^ T regulatory cells. J. Immunol..

[94] Adda D.H., Beraud E., Depieds R. (1977). Evidence for suppressor cells in Lewis rats’ experimental allergic encephalomyelitis. Eur. J. Immunol..

[95] Adda D.H., Beraud E., Depieds R. (1977). Suppressor cells in allergic encephalomyelitis. Ann. Immunol..

[96] Killen J.A., Swanborg R.H. (1982). Regulation of experimental allergic encephalomyelitis. Part 4. Further characterization of postrecovery suppressor cells. J. Neuroimmunol..

[97] Karpus W.J., Swanborg R.H. (1989). CD4^+^ suppressor cells differentially affect the production of IFN-gamma by effector cells of experimental autoimmune encephalomyelitis. J. Immunol..

[98] Hawiger D., Wan Y.Y., Eynon E.E., Flavell R.A. (2010). The transcription cofactor Hopx is required for regulatory T cell function in dendritic cell-mediated peripheral T cell unresponsiveness. Nat. Immunol..

[99] Josefowicz S.Z., Niec R.E., Kim H.Y., Treuting P., Chinen T., Zheng Y., Umetsu D.T., Rudensky A.Y. (2012). Extrathymically generated regulatory T cells control mucosal TH2 inflammation. Nature.

[100] Gianani R., Eisenbarth G.S. (2005). The stages of type 1A diabetes: 2005. Immunol. Rev..

[101] Atkinson M.A., Eisenbarth G.S., Michels A.W. (2014). Type 1 diabetes. Lancet.

[102] Makino S., Kunimoto K., Muraoka Y., Mizushima Y., Katagiri K., Tochino Y. (1980). Breeding of a non-obese, diabetic strain of mice. Jikken Dobutsu.

[103] Atkinson M.A., Leiter E.H. (1999). The NOD mouse model of type 1 diabetes: As good as it gets?. Nat. Med..

[104] Mullen Y. (2017). Development of the Nonobese Diabetic Mouse and Contribution of Animal Models for Understanding Type 1 Diabetes. Pancreas.

[105] Bruder D., Westendorf A.M., Hansen W., Prettin S., Gruber A.D., Qian Y., von Boehmer H., Mahnke K., Buer J. (2005). On the edge of autoimmunity: T-cell stimulation by steady-state dendritic cells prevents autoimmune diabetes. Diabetes.

[106] Lo D., Freedman J., Hesse S., Palmiter R.D., Brinster R.L., Sherman L.A. (1992). Peripheral tolerance to an islet cell-specific hemagglutinin transgene affects both CD4^+^ and CD8^+^ T cells. Eur. J. Immunol..

[107] Apostolou I., Von Boehmer H. (2004). The TCR-HA, INS-HA transgenic model of autoimmune diabetes: Limitations and expectations. J. Autoimmun..

[108] Mukhopadhaya A., Hanafusa T., Jarchum I., Chen Y.G., Iwai Y., Serreze D.V., Steinman R.M., Tarbell K.V., DiLorenzo T.P. (2008). Selective delivery of beta cell antigen to dendritic cells in vivo leads to deletion and tolerance of autoreactive CD8^+^ T cells in NOD mice. Proc. Natl. Acad. Sci. USA.

[109] Price J.D., Hotta-Iwamura C., Zhao Y., Beauchamp N.M., Tarbell K.V. (2015). DCIR2^+^ cDC2 DCs and Zbtb32 Restore CD4^+^ T-Cell Tolerance and Inhibit Diabetes. Diabetes.

[110] Petzold C., Riewaldt J., Koenig T., Schallenberg S., Kretschmer K. (2010). Dendritic cell-targeted pancreatic beta-cell antigen leads to conversion of self-reactive CD4(+) T cells into regulatory T cells and promotes immunotolerance in NOD mice. Rev. Diabet. Stud..

[111] Mukherjee G., Geliebter A., Babad J., Santamaria P., Serreze D.V., Freeman G.J., Tarbell K.V., Sharpe A., DiLorenzo T.P. (2013). DEC-205-mediated antigen targeting to steady-state dendritic cells induces deletion of diabetogenic CD8(+) T cells independently of PD-1 and PD-L1. Int. Immunol..

[112] Spiering R., Margry B., Keijzer C., Petzold C., Hoek A., Wagenaar-Hilbers J., van der Zee R., van Eden W., Kretschmer K., Broere F. (2015). DEC205^+^ Dendritic Cell-Targeted Tolerogenic Vaccination Promotes Immune Tolerance in Experimental Autoimmune Arthritis. J. Immunol..

[113] Wadwa M., Klopfleisch R., Buer J., Westendorf A.M. (2016). Targeting Antigens to Dec-205 on Dendritic Cells Induces Immune Protection in Experimental Colitis in Mice. Eur. J. Microbiol. Immunol..

[114] Caspi R.R., Roberge F.G., Chan C.C., Wiggert B., Chader G.J., Rozenszajn L.A., Lando Z., Nussenblatt R.B. (1988). A new model of autoimmune disease. Experimental autoimmune uveoretinitis induced in mice with two different retinal antigens. J. Immunol..

[115] Kamoi K., Martin-Granados C., Bobu C., Wikstro M.E., Degli-Esposti M.A., Steinman R.M., Forrester J.V. (2012). Anti-DEC205 Mediated Delivery of Self-Antigen to Dendritic Cell Restores Tolerance in Spontaneous EAU. Investig. Opthamol. Vis. Sci..

[116] Xu H., Chen M. (2016). Targeting the complement system for the management of retinal inflammatory and degenerative diseases. Eur. J. Pharm..

[117] Vanikar A. (2014). Transplantation tolerance; myth or reality?. J. Nephropathol..

[118] Salama A.D., Womer K.L., Sayegh M.H. (2007). Clinical transplantation tolerance: Many rivers to cross. J. Immunol..

[119] Saxena V., Li L., Paluskievicz C., Kasinath V., Bean A., Abdi R., Jewell C.M., Bromberg J.S. (2019). Role of lymph node stroma and microenvironment in T cell tolerance. Immunol. Rev..

[120] McCurry K.R., Colvin B.L., Zahorchak A.F., Thomson A.W. (2006). Regulatory dendritic cell therapy in organ transplantation. Transpl. Int..

[121] Morelli A.E., Thomson A.W. (2014). Orchestration of transplantation tolerance by regulatory dendritic cell therapy or in-situ targeting of dendritic cells. Curr. Opin. Organ Transpl..

[122] Tanriver Y., Ratnasothy K., Bucy R.P., Lombardi G., Lechler R. (2010). Targeting MHC class I monomers to dendritic cells inhibits the indirect pathway of allorecognition and the production of IgG alloantibodies leading to long-term allograft survival. J. Immunol..

[123] Ochando J., Ordikhani F., Jordan S., Boros P., Thomson A.W. (2020). Tolerogenic dendritic cells in organ transplantation. Transpl. Int..

[124] Ettinger M., Gratz I.K., Gruber C., Hauser-Kronberger C., Johnson T.S., Mahnke K., Thalhamer J., Hintner H., Peckl-Schmid D., Bauer J.W. (2012). Targeting of the hNC16A collagen domain to dendritic cells induces tolerance to human type XVII collagen. Exp. Derm..

[125] Obregon C., Kumar R., Pascual M.A., Vassalli G., Golshayan D. (2017). Update on Dendritic Cell-Induced Immunological and Clinical Tolerance. Front. Immunol..

[126] Marin E., Cuturi M.C., Moreau A. (2018). Tolerogenic Dendritic Cells in Solid Organ Transplantation: Where Do We Stand?. Front. Immunol..

[127] Thomson A.W., Metes D.M., Ezzelarab M.B., Raich-Regue D. (2019). Regulatory dendritic cells for human organ transplantation. Transpl. Rev..

[128] Bulatov E.K.S., dos Reis H.J., Palotás A., Venkataraman K., Vijayalakshmi M., Rizvanov A. (2016). Ubiquitin-Proteasome System: Promising Therapeutic Targets in Autoimmune and Neurodegenerative Diseases. Bionanoscience.

[129] Ziegler-Heitbrock L., Ancuta P., Crowe S., Dalod M., Grau V., Hart D.N., Leenen P.J., Liu Y.J., MacPherson G., Randolph G.J. (2010). Nomenclature of monocytes and dendritic cells in blood. Blood.

[130] Murphy K.M. (2013). Transcriptional control of dendritic cell development. Adv. Immunol..

